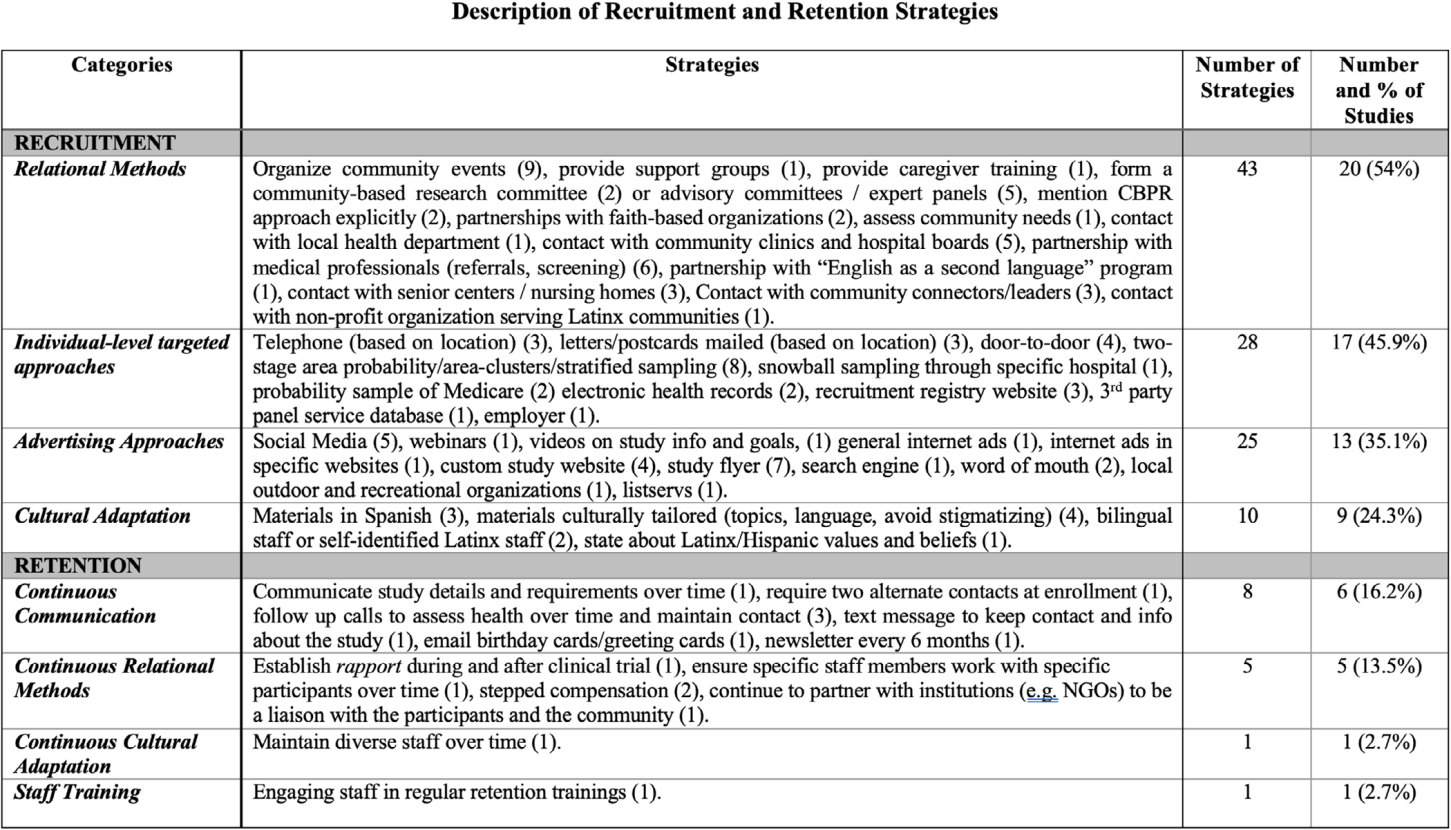# Mapping Strategies for Recruiting and Retaining US Hispanic/Latino Populations in AD/ADRD Clinical Trials and Longitudinal Research: A Scoping Review

**DOI:** 10.1002/alz70860_103981

**Published:** 2025-12-23

**Authors:** Sharon Sanz Simon, Isabel Solis, Melany Medina, Alejandra Morlett‐Paredes, Mirella Diaz‐Santos, Irving Vega, Serggio Lanata, Ignacia Arteaga Perez, Clara Vila‐Castelar, Maria P. P. Aranda, María M. M. Corrada, Jorge J. Llibre‐Guerra, Elena Portacolone, Sudha Seshadri, Yakeel T. Quiroz, Gladys E. Maestre, David X. Marquez, Christian R Salazar

**Affiliations:** ^1^ New Jersey Medical School, Rutgers University, Newark, NJ, USA; ^2^ Massachusetts General Hospital, Harvard Medical School, Boston, MA, USA; ^3^ Institute for Memory Impairments and Neurological Disorders, University of California, Irvine, Irvine, CA, USA; ^4^ University of California San Diego, San Diego, CA, USA; ^5^ David Geffen School of Medicine, University of California, Los Angeles, Los Angeles, CA, USA; ^6^ Michigan State University, Grand Rapids, MI, USA; ^7^ Memory and Aging Center, UCSF Weill Institute for Neurosciences, University of California, San Francisco, San Francisco, CA, USA; ^8^ University of California, San Francisco, San Francisco, CA, USA; ^9^ University of Southern California, Los Angeles, CA, USA; ^10^ University of California, Irvine, Irvine, CA, USA; ^11^ Washington University in St. Louis School of Medicine, St. Louis, MO, USA; ^12^ University of California San Francisco, San Francisco, CA, USA; ^13^ University of Texas Health San Antonio, San Antonio, TX, USA; ^14^ The University of Texas Rio Grande Valley School of Medicine, Brownsville, TX, USA; ^15^ Department of Kinesiology and Nutrition, University of Illinois Chicago, Chicago, IL, USA

## Abstract

**Background:**

Latinos represent the largest racial/ethnic minoritized group in the United States, yet their participation in Alzheimer's disease and related dementias (AD/ADRD) clinical trials and longitudinal research remains disproportionately low. This scoping review aims to identify patterns, highlight gaps, and provide actionable recommendations for recruitment and retention strategies targeting Latino communities in AD/ADRD clinical trials and longitudinal cohorts.

**Method:**

A comprehensive search of MEDLINE was independently conducted by two reviewers, and the reference lists of included studies were screened for additional sources. Four review authors performed screening and data extraction in parallel, ensuring quality through double‐checking and consensus. The search included studies published from 1990 to September 2024, focusing on: 1) recruitment and retention, 2) diverse Latino heritages, and 3) AD/ADRD, and neurocognitive disorders. Eligible studies included: 1) clinical trials or longitudinal cohorts, 2) Latino populations in the US or its territories, and 3) any clinical condition.

**Result:**

Of the 504 abstracts identified, 37 studies met the inclusion criteria, comprising 16 clinical trials and 21 longitudinal cohort studies. Among these, 31 (83.7%) reported recruitment strategies, 9 (21.6%) described retention strategies, and 6 studies (16.2%) reported no strategies at all. Recruitment strategies varied, with the most common approaches being: 1) relational methods (54.0%); 2) individual‐level targeted approaches (45.9%); 3) advertising (35.1%), and 4) cultural adaptations (24.3%). Retention approaches were less frequently reported, with the most common being: 1) continuous communication (16.2%); 2) continuous relational methods (13.5%); 3) cultural adaptation iterations (2.7%); and 4) underrepresented minoritized staff training (2.7%). Notable gaps included a limited focus on staff training and population‐based recruitment methods.

**Conclusion:**

While efforts to foster community partnerships and individual engagement are documented, fewer studies detail culturally and linguistically tailored approaches or strategies for sustained continuous communication and relationship‐building with stakeholders. Moreover, a critical gap exists in training research staff to work effectively with underrepresented populations. These deficiencies may exacerbate the underrepresentation of Latino communities in clinical research. Addressing these gaps and prioritizing culturally responsive strategies are critical areas for future research.